# Social Media as an Effective Provider of Quality-Assured and Accurate Information to Increase Vaccine Rates: Systematic Review

**DOI:** 10.2196/50276

**Published:** 2023-12-26

**Authors:** Rita-Kristin Hansen, Nikita Baiju, Elia Gabarron

**Affiliations:** 1 Department of Community Medicine, Faculty of Health Sciences, UiT The Arctic University of Norway Tromsø Norway; 2 Department of Education, ICT and Learning, Østfold University College Halden Norway; 3 Norwegian Centre for E-health Research Tromsø Norway

**Keywords:** social media, vaccines, vaccination, randomized controlled trials, information sources

## Abstract

**Background:**

Vaccination programs are instrumental in prolonging and improving people’s lives by preventing diseases such as measles, diphtheria, tetanus, pertussis, and influenza from escalating into fatal epidemics. Despite the significant impact of these programs, a substantial number of individuals, including 20 million infants annually, lack sufficient access to vaccines. Therefore, it is imperative to raise awareness about vaccination programs.

**Objective:**

This study aims to investigate the potential utilization of social media, assessing its scalability and robustness in delivering accurate and reliable information to individuals who are contemplating vaccination decisions for themselves or on behalf of their children.

**Methods:**

The protocol for this review is registered in PROSPERO (identifier CRD42022304229) and is being carried out in compliance with the Cochrane Handbook for Systematic Reviews of Interventions. Comprehensive searches have been conducted in databases including MEDLINE, Embase, PsycINFO, CINAHL (Cumulative Index to Nursing and Allied Health), CENTRAL (Cochrane Central Register of Controlled Trials), and Google Scholar. Only randomized controlled trials (RCTs) were deemed eligible for inclusion in this study. The target population encompasses the general public, including adults, children, and adolescents. The defined interventions comprise platforms facilitating 2-way communication for sharing information. These interventions were compared against traditional interventions and teaching methods, referred to as the control group. The outcomes assessed in the included studies encompassed days unvaccinated, vaccine acceptance, and the uptake of vaccines compared with baseline. The studies underwent a risk-of-bias assessment utilizing the Cochrane Risk-of-Bias tool for RCTs, and the certainty of evidence was evaluated using the GRADE (Grading of Recommendations Assessment, Development, and Evaluation) assessment.

**Results:**

This review included 10 studies, detailed in 12 articles published between 2012 and 2022, conducted in the United States, China, Jordan, Australia, and Israel. The studies involved platforms such as Facebook, Twitter, WhatsApp, and non–general-purpose social media. The outcomes examined in these studies focused on the uptake of vaccines compared with baseline, vaccine acceptance, and the number of days individuals remained unvaccinated. The overall sample size for this review was 26,286, with individual studies ranging from 58 to 21,592 participants. The effect direction plot derived from articles of good and fair quality indicated a nonsignificant outcome (*P*=.12).

**Conclusions:**

The findings suggest that, in a real-world scenario, an equal number of positive and negative results may be expected due to the interventions’ impact on the acceptance and uptake of vaccines. Nevertheless, there is a rationale for accumulating experience to optimize the use of social media with the aim of enhancing vaccination rates. Social media can serve as a tool with the potential to disseminate information and boost vaccination rates within a population. However, relying solely on social media is not sufficient, given the complex structures at play in vaccine acceptance. Effectiveness hinges on various factors working in tandem. It is crucial that authorized personnel closely monitor and moderate discussions on social media to ensure responsible and accurate information dissemination.

## Introduction

Vaccination stands as a success story, viewed both through a global health lens and from a developmental perspective. This intervention effectively prevents over 20 life-threatening diseases, contributing to the prevention of 2-3 million deaths worldwide annually [1]. Vaccination programs rank among the most crucial contributors to extended and healthier lives, preventing diseases such as measles, diphtheria, tetanus, pertussis, and influenza from escalating into fatal epidemics [1]. In certain instances, vaccines have played a decisive role in eradicating life-threatening diseases such as smallpox and poliomyelitis. We have witnessed a remarkable reduction of over 95% in the incidence of diseases such as diphtheria, tetanus, pertussis, mumps, and rubella [2]. Furthermore, vaccines play a crucial role in global health security by serving as vital tools in the fight against antimicrobial resistance [1]. Prophylactic use of vaccines not only decreases the prevalence of infectious diseases but also contributes to a reduction in the use of antibiotics. This pathway, in turn, leads to a desirable outcome by reducing the spread and emergence of antimicrobial resistance [2]. The World Health Organization (WHO) encapsulates this narrative by asserting that vaccination is the most beneficial health investment money can buy [1].

Despite undeniable successes and health care progress, a significant number of individuals, including 20 million infants annually, still lack adequate access to vaccines [1]. Global vaccination coverage has shown stagnation over the past few years, with progress stalling or even regressing in some countries [1]. There is a noticeable divide in attitudes, with higher support observed in South Asia, South America, and Africa, but lower support noted in Europe, Russia, and North America [3].

According to the European Centre for Disease Prevention and Control (ECDC), a significant challenge we face is resistance in the population against vaccination, despite the established safety and effectiveness of vaccines [4]. The ECDC highlights a possible explanation that we might have grown accustomed to the benefits of vaccination. The collective memory of the devastating consequences of certain diseases may be weakening, particularly in regions where vaccine-preventable diseases have become rarer, especially in the Northern parts of the world [4]. Thus, there is a crucial need for the communication of accurate scientific facts to empower both policy makers and the public to make informed choices [4]. Social media has the potential to play a pivotal role in facilitating this communication and mitigating vaccine hesitancy [5].

More than three-fifths of the world’s population (61%) are utilizing some form of social media [6], and the popularity of these platforms continues to grow [7]. As of the beginning of 2023, the 5 most widely used social media platforms are Facebook (Meta Platforms, Inc.), YouTube (Google LLC), WhatsApp (Meta Platforms, Inc.), Instagram (Meta Platforms, Inc.), and WeChat (Tencent) [8]. The evolving reach of social media across diverse demographics can render it an effective information provider for increasing vaccine rates, provided it is used wisely and informatively. Previous research has already indicated that using various social media–based promotion methods could effectively enhance immunization coverage rates [9-11].

This review aims to investigate the potential utilization of social media, assessing its scalability and robustness in delivering accurate and reliable information to individuals making decisions about receiving vaccinations for themselves or on behalf of their children.

## Methods

### Review Guidelines and Protocol Registration

This review adhered to the guidelines outlined in the Cochrane Handbook for Systematic Reviews of Interventions, version 6.3, 2022 [12]. The reporting of the systematic review was conducted in accordance with the PRISMA (Preferred Reporting Items for Systematic Reviews and Meta-Analyses) guidelines (Multimedia Appendix 1) [13]. The review protocol was registered in PROSPERO on March 14, 2022, with the ID CRD42022304229 [14]. Searches were conducted from the year 1946 to June 29, 2023.

### Search Strategy and Selection of the Literature

We conducted searches for publications of randomized controlled trials (RCTs) using keywords related to social media and vaccination campaigns. Journal articles were sought in databases including MEDLINE, Embase, PsycINFO, CINAHL (Cumulative Index to Nursing and Allied Health), and CENTRAL (Cochrane Central Register of Controlled Trials). Additionally, Google Scholar was searched, and the first 200 hits were assessed for eligibility criteria. Searches were also conducted in the International Clinical Trials Registry Platform (ICTRP) and ClinicalTrials.gov for ongoing studies. The reference lists of systematic reviews and other relevant publications were checked for studies that might not have been identified in the initial database searches. Gray literature was consulted by conducting searches on Google (Alphabet Inc.) using terms such as social media, vaccine, vaccination, and randomized trial. For the complete search strategy, please refer to Multimedia Appendix 2.

The identified references were uploaded to EndNote (Clarivate Plc.) version 20.3 [15] and Rayyan (Rayyan Systems Inc.) [16]. Two reviewers (RKH and EG) independently participated in the selection of studies. Any disagreements were resolved through discussion with a third reviewer (NB).

### Inclusion and Exclusion Criteria

Inclusion and exclusion criteria were based on PICO (*p*opulation, *i*ntervention/exposures, *c*omparison, *o*utcomes) elements and are listed in Textbox 1.

**Inclusion and exclusion criteria.**

**1. Inclusion Criteria**

- Population: the public in general (ie, adults, children, and adolescents).
- Intervention/exposures: social media (ie, a platform that provides the opportunity to share information between the provider of information and the receiver of the information; also described as a 2-way communication) [17]. This meant that concepts that were named web-based intervention, internet-based intervention, eHealth, or interactive health communication were included.
- Comparison: anything besides social media (ie, traditional information, traditional education, or no comparisons were eligible criteria for the control/comparison group).
- Outcomes: the effect of social media intervention on the number of vaccinations or vaccination rates [14] (ie, days unvaccinated, vaccine acceptance, or uptake of vaccines compared with baseline).
- Study design: randomized controlled trials.
- Other criteria: any type of vaccine, for example, a vaccine against human papillomavirus, seasonal influenza, tetanus, diphtheria, pertussis, or COVID-19.

**2. Exclusion Criteria**

- Studies that did not meet all inclusion criteria or were published in languages other than English, Norwegian, Swedish, or Danish were excluded from the review.

### Risk-of-Bias Assessment and Certainty on Evidence

Two reviewers (RKH and EG) independently assessed the risk of bias using the Cochrane Risk-of-Bias Tool for RCTs [18], and the certainty of evidence was evaluated using the GRADE (Grading of Recommendations Assessment, Development, and Evaluation) assessment [19]. Any disagreements were resolved through discussion with a third reviewer (NB).

### Data Extraction

A single reviewer (RKH) conducted the data extraction, and a second reviewer (EG) verified the accuracy and completeness of the extracted data. The data extraction focused on the categorization of studies and how to potentially pool the results. The extracted information included the following: (1) bibliographic information (authors, date, title, and country); (2) study characteristics (duration of the study, study setting, study design, loss to follow-up, and type of vaccines); (3) population (average/mean age, gender, sample size, ethnicity, and socioeconomic status); (4) intervention (type of social media); (5) comparison or control group; (6) outcome/measurement (days unvaccinated, vaccine acceptance, and uptake of vaccines compared with baseline); and (7) outcomes (vaccine rate and the authors’ conclusions from their studies).

### Data Analysis

To visualize the effect of the intervention, an effect direction plot was created following the guidance outlined in the Cochrane Handbook on alternative synthesis methods. *P* values were calculated using GraphPad (GraphPad Software Inc.) [20] to test the probability of the null hypothesis. The number of positive and negative effect direction arrows was counted for each outcome domain. All inconsistent effect directions were excluded from this sign test, given the calculation of a 1-tailed *P* value for each outcome domain.

## Results

### Study Selection

**Figure 1:**
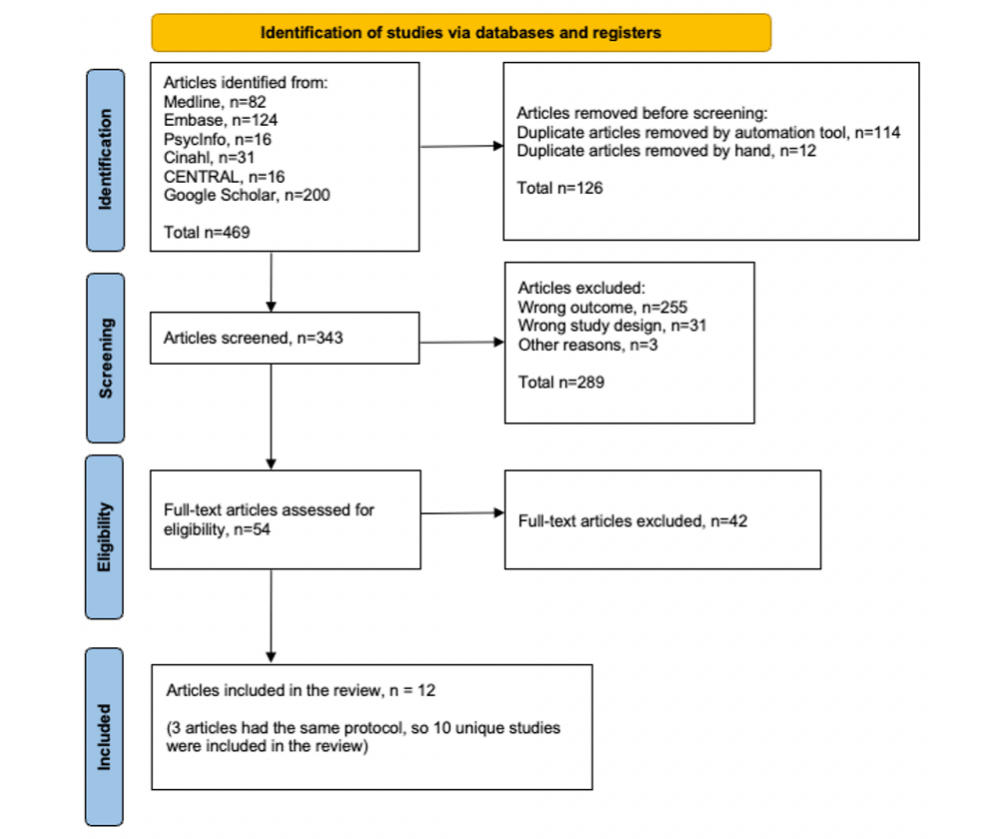
PRISMA (Preferred Reporting Items for Systematic Reviews and Meta-Analyses) flowchart.

A total of 469 hits were identified, and 126 duplicate articles were removed. The remaining 343 articles were assessed for relevance, with 289 being excluded due to incorrect outcomes, study design, or other reasons. Subsequently, 54 articles were eligible for full-text screening, of which 42 articles were excluded (refer to Multimedia Appendix 3 for an overview of the rejected articles and the reasons for their rejection; also see [21-62]). Ultimately, the result of these screenings resulted in the inclusion of 12 articles in this systematic review. Notably, 3 of these articles originated from the same protocol and were considered as 1 study. Refer to Figure 1 for a visual representation of the screening process [13].

### Study Characteristics

This review encompasses 10 distinct studies presented across 12 articles. Notably, 3 articles [63-65] explored 3 different outcomes from the same study protocol [66]. The studies included in this review span from 2012 [67] to the most recent in 2022 [68]. Table 1 presents an overview of the included articles.

**Table 1:** Characteristics of included articles (n=12).

Authors	General characteristics	Population	Intervention/type of social media	Comparison/control group	Outcome/measurement	Risk of bias^a^
Daley et al [63]^b^	Study design: RCT^c^ Vaccine type: vaccines in general Colorado, United States	Member of KPCO^d^ Recruited during pregnancy Age: 31.6 (SD 4.4) years Only females N=1093	A study website with vaccine information and social media components (VSM arm).	A website with vaccine information only Usual care	Vaccine acceptance Change in parental vaccine attitudes over time by baseline degree of vaccine hesitancy.	Good quality
Glanz et al [64]^b^	Study design: RCT Vaccine type: vaccines in general Colorado, United States	Member of KPCO Recruited during pregnancy Age: 31.6 (SD 4.3) years Only females N=1093	Multidirectional communication model: (1) website developers created and presented content to users; (2) users created content and interacted with website developers; and (3) users interacted with each other and shared information.	A website with vaccine information only Usual care	Days unvaccinated From birth to age 200 days	Good quality
O’Leary et al [65]^b^	Study design: RCT Vaccine type: influenza and Tdap^e^ Colorado, United States	Women in the third trimester of pregnancy integrated into KPCO Age: 32 (SD 4.5) years Only female N=1093	A website with vaccine information and interactive social media components. Included a blog and a discussion forum and an “Ask a question” portal.	A website with vaccine information only Usual care	Uptake of vaccines compared with baseline Receipt of influenza and Tdap vaccines among pregnant women.	Good quality
Liao et al [69]	Study design: RCT Vaccine type: childhood SIV^f^ China, Hong Kong	Mothers of child(ren) aged 6-72 months Age: N/A^g^ Only female N=365	WhatsApp weekly vaccination reminders WhatsApp discussion group	No intervention	Uptake of vaccines compared with baseline SIV uptake in children	Good quality
Zhang et al [70]	Study design: online survey experiment Vaccine type: influenza, HPV^h^, MMR^i^, Tdap, Zika United States	Adults recruited from Dynata^j^ Age: 41.13 (SD 13.42) years 50.2% female N=1198	Mock Twitter page and fact-checking labels: the treatment groups added a simple fact-checking label below the misinformation message, which consisted of a red warning sign, a falsification message, and a source logo.	A tweet consisting of a picture of a bottle of a specific vaccine and a misinformation claim only.	Vaccine acceptance Vaccine attitudes	Good quality
Ugarte et al [68]	Study design: RCT Vaccine type: COVID-19 United States	Adults recruited from online advertisements Age: 39.02 (SD 10.90) years 78.7% female N=108	Online support community of peers trained in behavior change science Facebook groups	Online community without peer leaders	Vaccine acceptance Vaccine uptake	Good quality
Abdel-Qader et al [71]	Study design: RCT Vaccine type: COVID-19 Jordan	Adult population who were reluctant or resistant to the COVID-19 vaccine Age: 18-64 years 56.1% female N=320	Pharmacists-physicians collaborative coaching intervention was delivered to active group participants over 2 months through Facebook live sessions.	The control group did not receive intervention	Uptake of vaccines compared with baseline The proportion of hesitancy and resistance to a COVID-19 vaccine The proportion of patients vaccinated	Fair quality
Brandt et al [72]	Study design: a controlled, quasi-experimental mixed methods study Vaccine type: HPV United States	College students: two undergraduate classes at a public university in the southeast region of the United States Age: 21.6 (SD 2.2) years Female n=47, male n=11 N=58	Facebook private group posts Weekly emails	Behavioral weight gain prevention intervention (Healthy Weight) Classes were randomized to receive either an HPV vaccination awareness intervention or a behavioral weight gain prevention intervention (Healthy Weight; control). Each group served as the control for the other group, allowing for simultaneous intervention comparisons.	Vaccine acceptance HPV vaccination status and intentions HPV vaccination knowledge	Fair quality
Lau et al [67]	Study design: RCT Vaccine type: SIV Australia	University students and staff Age: 26.2 (SD 9.07) years 57% female N=742	Healthy.me: a web-based personally controlled health management system on the uptake of seasonal influenza vaccine and primary care service utilization among university students and staff.	6-month waitlist	Uptake of vaccines compared with baseline Uptake of seasonal influenza vaccine	Fair quality
Chodick et al [73]	Study design: RCT Vaccine type: HPV Israel	MHS^k,l^ members who were mothers to 14-year-old daughters in the 2019 school year (who were born between October 2004 and December 2005) Age: 44.6 (SD 5.2) years Only female N=21,592	Facebook Targeted campaign	The control group (20%) did not receive targeted campaign messages.	Uptake of vaccines compared with baseline HPV immunization history among the eighth-grade daughters of the study participants	Poor quality
Ortiz et al [74]	Study design: online survey experiment Vaccine type: HPV United States	Adolescents who had not completed the HPV vaccine series Age: 15.6 (SD 1.68) years 60.2% female N=108	Facebook: providing relevant health information from a credible health source via a commonly used social media platform.	No intervention, just another email to complete a second survey questionnaire.	Vaccine acceptance Improve adolescents’ knowledge about vaccination against HPV	Poor quality
Osborne et al [75]	Study design: RCT Vaccine type: SIV United States	Undergraduates at a large midwestern public university Age: >18 years 70% female N=702	Twitter: following a Twitter account that posted near-daily tweets (1.24 tweets per day) promoting flu vaccination. In addition to direct tweet exposure, campaign engagement was incentivized with prize raffle entries. For each month of the study, an intervention group member could receive 1 raffle entry (up to 7 over the study) by retweeting 1 of the promotional tweets, or by constructing their own tweet containing a hashtag that was unique to the campaign.	Following a Twitter account that tweeted no content.	Uptake of vaccines compared with baseline Vaccine rates	Poor quality

^a^Risk of bias was assessed by RKH and EG using the Cochrane Risk-of-Bias Tool for Randomized Controlled Trials.

^b^Daley et al [63], Glanz et al [64], O’Leary et al [65] belong to the same protocol in ClinicalTrials.com 24.

^c^RCT: randomized controlled trial.

^d^KPCO: Kaiser Permanente Colorado.

^e^Tdap: tetanus, diphtheria, pertussis.

^f^SIV: seasonal influenza vaccine.

^g^N/A: not applicable.

^h^HPV: human papillomavirus.

^i^MMR: measles, mumps, and rubella.

^j^Dynata (Research Now) maintains a large panel of American adults recruited via verified sources, uses multiple layers of authentication, and periodically invites the panel to take part in studies.

^k^State-mandated health organization in Israel (MHS).

^l^MHS: Maccabi Healthcare Services.

### Risk-of-Bias Assessment in Studies

#### Population

**Figure 2:**
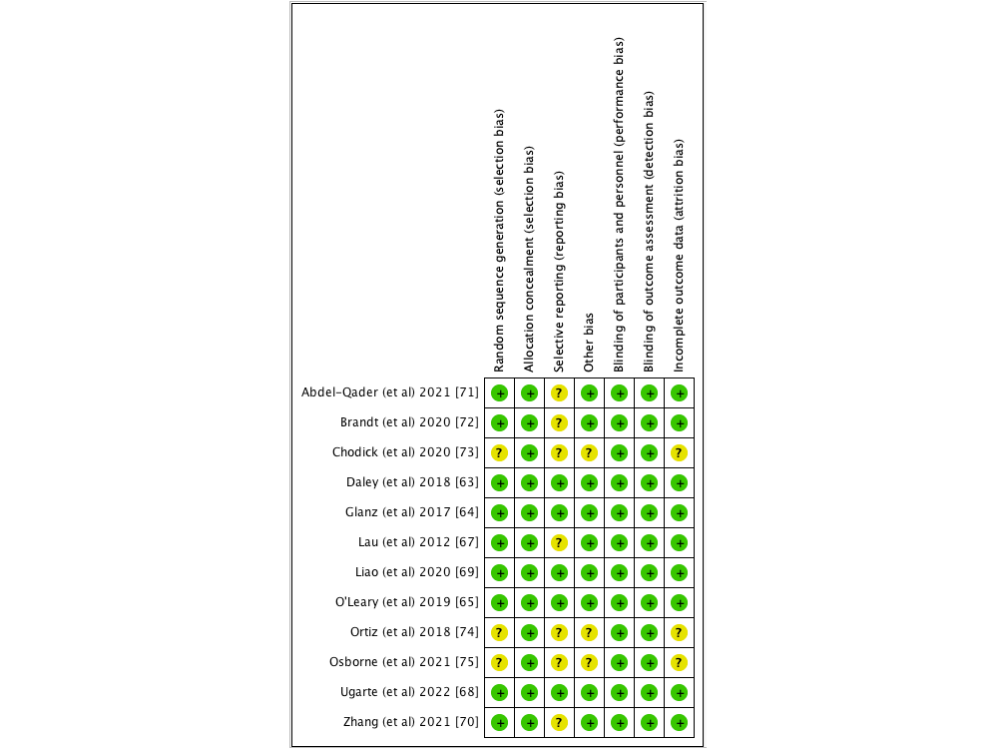
Risk of bias summary: risk of bias for each included study.

A total of 26,286 individuals participated in the studies included in this review. Among the 12 included articles, 5 [63-65,69,73] focused specifically on females only (including pregnant women, mothers of adolescent girls, or mothers of toddlers). In the remaining 7 articles, both genders were represented. As many as 8 out of the 12 studies were conducted in the United States [63-65,68,70,72,74,75], with 3 of these in the same setting [63-65]. The remaining studies were conducted in China [69], Jordan [71], Australia [67], and Israel [73]. Refer to Table 1 for details. See Figures 2 [63-65, 67-75] and 3 [76] for the risk-of-bias summary and risk-of-bias item, respectively.

**Figure 3:**
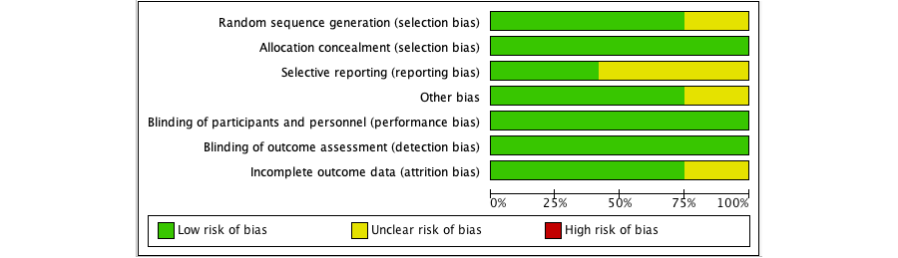
Risk of bias item presented as percentages across all included studies.

#### Intervention

The most commonly used social media platform as an intervention in the included studies (n=5) was Facebook [68,71-74]. A study of good quality [68] utilized Facebook groups to assess the efficacy of a peer-led intervention aimed at promoting requests for COVID-19 vaccine information among essential workers. Two studies rated as fair quality in the risk-of-bias assessment used Facebook as a platform to explore COVID-19 vaccine hesitancy among residents of Jordan (n=320) [71] and for health promotion among 2 undergraduate classes at a public university in the southeastern region of the United States (n=58) [72]. The other 2 studies [73,74] utilizing Facebook as an intervention were rated as poor-quality studies, both investigating measures to increase vaccine rates or knowledge of human papillomavirus (HPV).

Two studies [70,75] utilized Twitter (n=2). One of these studies was rated as good quality in the risk-of-bias assessment. This study examined a mock Twitter page and investigated the effect of fact-checking social media vaccine misinformation [70]. The other study that examined Twitter was rated as poor quality. In this trial, the intervention group members followed a Twitter account that posted daily tweets promoting flu vaccination [75].

One study utilized WhatsApp [69]. The intervention involved weekly vaccination reminders and a WhatsApp discussion group, described as a time pressure and social networking intervention. This study was rated as good quality.

Four articles did not specify general-purpose social media platforms for their interventions. The 3 articles [63-65] from the same study protocol described a study website with vaccine information and social media components (blog, discussion forum, and a chat room). All 3 articles, rated as good quality, reported positive effects of the intervention on the outcome. They demonstrated significant results concerning the exposure of the website with vaccine information and social media components on the 3 different outcomes. Additionally, the personal web-based controlled health management system Healthy.me was used in a university setting to manage the uptake of seasonal influenza vaccine and primary care services [67]. This study was rated as *fair quality* in the risk-of-bias assessment in this review. It reported a dose-response effect, indicating that increased use of the intervention was associated with higher rates of vaccination and more visits to the health service provider [67].

#### Control Group/Comparison

A total of 6 articles [63-65,68-70], considered of good quality, provided descriptions of either the control group or the comparison group. The 3 articles [63-65] belonging to the same study randomly assigned participants into 3 groups (3:2:1): a website with vaccine information and interactive social media components; website with vaccine information only; or the group receiving usual care. In 1 study [68] the control group consisted of an online community without peer leaders. Another study [69] described the control group as “no intervention.” In the last study within the category of good quality, participants were randomized into 3 groups (5:2:2): a control group, a social networking intervention without time pressure, and a social networking intervention with time pressure. The control group in this study involved distributing misinformation [70]. Despite ethical concerns regarding the dissemination of false information, this study design offers a valuable opportunity to compare the control group with the active group. Additionally, it provides a basis for comparisons between studies.

In 1 [71] of the 3 studies considered to be of *fair quality*, the control group was not adequately described. The study mentioned the existence of a control group without providing details, making the comparison of the groups somewhat unclear, as the impact on the control participants was not specified. In another study, college students were assigned to 2 groups: 1 receiving HPV vaccination awareness and the other a behavioral weight gain prevention intervention [72]. In the context of the healthy weight study, the HPV vaccination awareness group served as the control [72]. In the last study within the *fair quality* category, a waitlist was used as the control group [67]. The article did not provide further details about this group, leaving it unclear as to whether participants randomized into this group received any form of intervention.

In the *poor-quality* category, 1 study [74] described that the control group did not receive any intervention, but only an email to complete a second survey. Another study [73] characterized the control group as a Facebook group that received no targeted messages. The final study in this category [75] randomized participants into a group assigned to a control Twitter account, which tweeted no content.

### Intervention Outcomes

The outcomes of the 12 included articles were categorized according to the prespecified outcomes. Six studies [65,67,69,71,73,75] reported on the uptake of vaccines compared with baseline, 5 [63,68,70,72,74] assessed vaccine acceptance, and 1 [64] analyzed the days unvaccinated.

Four studies [65,67,69,71] of *good or fair quality* examined the uptake of vaccines compared with baseline. One of the studies [69] reported no difference between the 2 study groups. The other 3 studies reported a positive effect from the intervention: coaching through Facebook live sessions was found to be effective in reducing COVID-19 vaccine hesitancy [71]; personal health management system had a small but significant effect on influenza vaccination rates [67]; and web-based interventions, with and without social media components, showed higher uptake rates of the influenza vaccine in pregnant women receiving the intervention [65]. The 2 studies [73,75] of poor quality reported no differences in vaccination outcomes between groups.

Five studies investigated vaccine acceptance as the outcome. Four of them [63,68,70,72], of good and fair quality, reported that the intervention had a positive impact on vaccine acceptance. The fifth study [74], of poor quality, reported that the Facebook intervention had a positive effect on vaccine knowledge and acceptance. For a visualization of the effect direction, see Table 2.

**Table 2:** Effect direction plot.^a,b^

Study	Study design	Days unvaccinated^c^	Uptake of vaccines^d^	Vaccine acceptance^d^
*Daley et al [63]*	*Randomized controlled trial*	N/A^e^	N/A	▲^f^
*Glanz et al [64]*	*Randomized controlled trial*	▲	N/A	N/A
*O’Leary et al [65]*	*Randomized controlled trial*	N/A	▲	N/A
*Liao et al [69]*	*Randomized controlled trial*	N/A	◄►^g^	N/A
*Zhang et al [70]*	*Online survey experiment*	N/A	N/A	▲
*Ugarte et al [68]*	*Randomized controlled trial*	N/A	N/A	▲
Abdel-Qader et al [71]	Randomized controlled trial	N/A	▲	N/A
Brandt et al [72]	Controlled quasi-experimental mixed methods	N/A	N/A	▲
Lau et al [67]	Randomized controlled trial	N/A	▲	N/A

^a^Study design: assessed as a randomized controlled trial.

^b^Italicized entries indicate a low risk of bias; nonitalicized entries indicate some concerns.

^c^Number of trials or experiments must be ≥2, and so, it was not possible to calculate the *P* value for the outcome “Days unvaccinated.”

^d^Sign test for positive effect direction (1-tailed): *P*=.13 for both uptake of vaccines and vaccine acceptance.

^e^N/A: not applicable.

^f^Positive health impact.

^g^No change/mixed effects/conflicting findings.

### Certainty of the Evidence

Because of variations in outcome measurement and reporting among the included studies, pooling the data across studies to generate a single-effect estimate was not possible. However, to provide a systematic and transparent assessment of the certainty of evidence, we performed a GRADE assessment based on 5 GRADE domains to judge our certainty in the studies. The certainty of evidence was influenced by *methodological limitations or risk of bias, indirectness, imprecision, inconsistency,* and *the likelihood of publication bias within the domains* [19]. The grading results indicate that there is a reason to have less confidence in the effect estimate. The GRADE assessment for the outcomes is presented in Table 3.

**Table 3:** Certainty of the evidence.

Outcome	Effect	Number of participants (studies)	Certainty of the evidence (GRADE^a^)	Comment
Uptake of vaccines assessed with Facebook, Healthy.me, WhatsApp, a website with vaccine interactive social media components, and Twitter (follow-up: mean 9 months)	Three studies showed a positive effect on the outcome [65,67,71]; 3 other studies did not show any effect [69,73,75]	24,799 (6 randomized controlled trials)	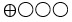 (very low^b^^,^^c,d^)	We have very little confidence in the effect estimate: the true effect is likely to be substantially different from the estimate of effect.
Vaccine acceptance was assessed with Facebook, a website with vaccine information and social media components, YouTube, and Twitter (follow-up: mean 9 months)	The studies showed a positive effect on the outcome [63,68,70,72,74]	2565 (5 randomized controlled trials)	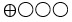 (very low^e^^,^^f,g^)	We have very little confidence in the effect estimate: the true effect is likely to be substantially different from the estimate of effect.
Days unvaccinated assessed with a website with vaccine information and social media components (follow-up: 36 months)	The study showed a positive effect on the outcome [64]	1093 (1 randomized controlled trial)	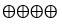 (high^h,i,j^)	We are very confident that the true effect lies close to that of the estimate of the effect.

^a^GRADE: Grading of Recommendations Assessment, Development, and Evaluation.

^b^Two [73,75] of 6 studies were rated *low quality*, 2 studies [67,71] were rated *fair quality*, and 2 [65,69] were rated *good quality* in the risk-of-bias assessment. Two studies [67,71] were rated as having an *unclear* risk of bias due to insufficient information on the domain *Selective reporting*. Two studies [73,75] were rated as having an unclear risk of bias in 4 domains (*Random sequence generation, Selective reporting, Other bias, and Incomplete outcome data*).

^c^The effect direction plot shows that 3 [65,67,71] of 6 studies reported a positive impact on the outcome (uptake of vaccines). Three of the studies [69,73,75] reported that the intervention had no effect on the outcome. The measures used in each study vary, so this made comparison of the studies difficult.

^d^The total number of participants in these 6 studies was 24,799. Four studies [65,67,69,73] reported CI, and all of them, except from 1 [73], reported wide intervals. Three of the CIs [65,67,73] were significant (*P*=.02 and *P*=.03 [73], *P*=.008 [67], and *P*=.01 [65]).

^e^One of 5 studies [74] was rated as having *low quality* in the risk-of-bias assessment. One study was rated as having *fair quality* [72], and 3 were rated as having *good quality* [63,68,70]. Two studies [70,72] were rated as having an *unclear* risk of bias in the domain *Selective reporting*, which is due to insufficient information to make a judgment.

^f^The effect direction plot shows that all 5 studies included [63,68,70,72,74] reported a positive impact on the outcome (vaccine acceptance). The measures used in each study vary, so this makes it difficult to compare the studies.

^g^The total number of participants was 2565. One study had n<60 [72]. Two studies reported CI. One was narrow and significant [63] and the other was wide and not significant [68].

^h^This study [64] was rated as *good quality* in the risk-of-bias assessment.

^i^The web-based vaccine information had a positive effect on parental vaccine behaviors.

^j^This study had 1093 participants. The CI reported between the active group and the control group was significant.

## Discussion

### Summary of the Main Results

Considering a health care system under pressure, it is crucial to explore how existing information channels can be leveraged to optimize the available resources within the health care system. In our review, we identified 12 randomized controlled trials (RCTs) that utilized social media to enhance vaccination rates. Although each of the studies contributes value by demonstrating positive results using various forms of social media to increase vaccine rates, the overall evidence remains limited.

### Should Vaccines Be Promoted Through Social Media?

As this review demonstrates, social media interventions have the potential to enhance knowledge about vaccines and increase the willingness to get vaccinated for oneself and one’s children. Previous research on vaccine hesitancy and behavior change theory–based social media interventions has also indicated this positive effect [5].

Robichaud et al [21] stated that there is an opportunity for public health organizations to actively engage in promoting factual and useful health messages regarding the benefits of vaccination using social media. Even 8 years later, D’Souza et al [77] investigated YouTube as a source of medical information on the COVID-19 virus disease. The authors advocated for information materials from official health agencies to disseminate valid and informative information to the public [77]. They also suggest that social media should be monitored by established health care personnel to maintain the platforms with fact-based knowledge on health issues and ensure that misleading and harmful information is not spread [77].

Previous research indicates that direct communication between health care personnel and the public is a factor that reduces vaccine concerns and might improve vaccine uptake [78]. Therefore, the use of social media platforms by health care personnel to enhance meaningful dialog regarding vaccine acceptance is encouraged [78]. This statement aligns with the findings in our review, where 3 studies [67,69,71] underscored the importance of active involvement from health care personnel in settings where health issues are communicated via social media platforms. It appears to be of great importance that health professionals, assuming roles such as informants, moderators, and effective discussion partners, play a role in distributing accurate and fact-based information on social media platforms. Mothers whose daughters have completed the vaccine program are considered effective representatives in influencing vaccine programs, as assessed by Buller et al [22]. Liao et al [69] stated that online information effectively promotes mothers’ self-efficacy to vaccinate their children against seasonal influenza. Nevertheless, the authors highlight that the active involvement of health professionals in online discussions is important in shaping positive discussions about vaccinations. Abdel-Qader et al [71] concluded that coaching by pharmacists and physicians through Facebook groups is effective in reducing rates of COVID-19 vaccine resistance and hesitancy. Ugarte et al [68] concluded that colleague guidance in the form of peer-led online Facebook groups can be useful for disseminating health information to help combat COVID-19 vaccine hesitation among essential health care workers.

### Implication for Practice and Future Research

The WHO estimates a projected shortfall of 10 million health workers by 2030, with the majority occurring in low- and lower-middle-income countries [79]. Health workforce shortages and the changing health needs of the public are contexts where digital transformation can offer unique opportunities [80]. Given the existing shortage of health personnel and the increasing burden on those who remain, it will be crucial to enhance the efficiency of those who will carry out health work in the future.

It is imperative to conduct further research on the mechanisms at play on social media with the aim of intervening in health issues such as vaccination. More systematic studies are needed to investigate how commercial social media platforms can effectively influence vaccination rates, allowing the results to be generalized to other settings and, potentially, to address other health issues. Digging deeper into specific issues, such as populations’ vaccine attitudes, would be significant for implementing timely interventions aimed at averting adverse public health consequences [81].

According to this review, there is a need to further explore which populations are most receptive to this type of intervention. Additionally, it is important to uncover the main features and characteristics of the most effective social media campaigns for vaccination.

### Trust, Transparency, and Framing the Content

When creating a social media intervention, establishing trust between the target population and the authorities and health care personnel is crucial [23,82]. Additionally, several other factors merit consideration: providing information on both risks and benefits, and acknowledging the concerns of the audience are essential components. Avoiding scientific jargon is imperative, and it is crucial to be transparent about funding sources. Referencing all sources of health information is equally important, along with providing quick responses and tailored personalized information [23]. It is essential to recognize that vaccine hesitancy is a complex phenomenon, not solely rooted in a deficit of comprehension. Vaccine hesitancy encompasses multifaceted considerations, including religious beliefs, safety concerns, low confidence in governments, and a range of other factors [83-85]. Recognizing this diversity of perspectives is crucial when formulating effective strategies to address and mitigate vaccine hesitancy within communities [82].

Previous research has shown a high prevalence of vaccine-related misinformation on social media [86], leading to vaccine hesitancy [83]. It is suggested that including fact-checking labels on posts containing misinformation can make viewers more favorable toward vaccines [70]. Designing, building, and evaluating theory-driven social media platforms aimed at making intervention recipients feel more comfortable about vaccines are suggested in the literature [23]. Additionally, monitoring by experts such as nurses, doctors, and other health care providers is recommended.

To influence the vaccine decision-making process, key factors include the source delivering the information, the network structure, and the framing of the information [87]. Similar findings are evident in other studies on this topic. For instance, a study revealed that vaccine-critical websites and blogs negatively impact the intention to vaccinate [78]. Moreover, even brief exposure to vaccine-critical websites increases beliefs in vaccine risk and hesitancy [88]. The question of framing the content of vaccine information becomes crucial in the construction of social media interventions. Lee et al [89] explored media design and choice for promoting HPV vaccination online, highlighting that the content itself plays a vital role in promoting health. They reference previous studies that describe messages emphasizing the negative consequences of neglecting recommended behavior, known as loss-framed messages, as more effective than the opposite kind of messages, namely, gain-framed messages [89].

### Strengths and Limitations

We acknowledge the presence of several limitations in this review. Language limitations were a factor, and as a result, we may have overlooked relevant studies published in other languages. It is possible that we did not identify all studies eligible for this review, but the likelihood of this is considered minor. Additionally, there may be studies published after the conclusion of the searches for this review.

Several limitations are associated with the included studies in this review. The level of heterogeneity was notably high, which limits the potential for quantitative comparisons in a meta-analysis and the ability to conduct subgroup analyses. This heterogeneity arises from divergent data across study populations, varied data collection methods, differences in exposures and outcomes assessed, and diverse applied methodologies. The utilization of social media platforms also varied significantly, with some studies describing platforms that cannot be directly generalized to other conditions. Furthermore, some studies were assigned a high risk of bias by the review members. The included studies also vary significantly in size, ranging from the smallest with 58 randomized participants to the largest with 21,592 participants. Summarizing the material available through this review, considering both the benefits and limitations of using social media as a means of communication for distributing vaccination information, is challenging due to these reasons. Additionally, the studies included are context specific, further complicating a comprehensive summary.

The distribution of the studied population is skewed, with a notable focus on women in a large portion of the studies. It is conceivable that a more balanced gender distribution might have yielded different results in some of the studies. Exploring how gender influences the receptivity of information distributed through social media is of significant importance and interest. Understanding whether there are differences in the way messages should be adapted to different gender categories could provide valuable insights. It is noteworthy that studies focusing on women were exclusively conducted in developed countries, and therefore, the results may not be readily generalized beyond these settings. In these environments, where there is a high probability that women’s decisions carry weight within a family considering the advantages and disadvantages of vaccination, the findings may be context specific. In developing countries, the situation can differ, and men’s voices may hold more influence. In cultures where gender roles strongly shape knowledge and acceptance of vaccination, it is crucial to consider these dynamics when planning how to effectively reach participants in a vaccination program.

Given that blinding was not applicable in the included studies, the domains related to this aspect were not taken into account during the GRADE assessment. Despite this, all 3 outcomes were graded, including the outcome assessed by only 1 study. Confidence in the evidence was primarily downgraded due to heterogeneity between the studies and concerns related to study designs. Two of the outcomes were downgraded due to the low quality of the studies as assessed in the risk-of-bias assessment, imprecision stemming from nonsignificant confidence intervals, and inconsistency in the varying forms of reporting results across different studies. Overall, the body of evidence is graded as low, indicating that the results must be interpreted with caution.

### Conclusions

This review underscores the substantial and untapped potential associated with using social media as a communication channel for health issues. With a strategic understanding of how to harness these mechanisms effectively, social media has the potential to reach a wide audience rapidly and in a cost-effective manner. Social media, when used as a supplementary promotional channel, can serve as an instrument for transmitting information that has the potential to increase vaccination rates in a population. However, the effectiveness of these tools relies on authorized personnel closely monitoring and moderating discussions. Numerous studies have explored how social media contributes to increased vaccine resistance. However, there is a pressing need for more knowledge on how social media can be optimally utilized to enhance vaccination rates in a population.

## Appendix

Multimedia Appendix 1 PRISMA (Preferred Reporting Items for Systematic Reviews and Meta-Analyses) checklist.

Multimedia Appendix 2 Search strategy.

Multimedia Appendix 3 Excluded studies examined in full text, and reasons for their exclusion.

## Abbreviations

CENTRAL: Cochrane Central Register of Controlled Trials
CINAHL: Cumulative Index to Nursing and Allied Health
ECDC: European Centre for Disease Prevention and Control
HPV: human papillomavirus
ICTRP: International Clinical Trials Registry Platform
KPCO: Kaisers Permanente Colorado
MMR: measles, mumps, and rubella
PCHMS: Personal Control Health Management System
PICO: population, intervention/exposures, comparison, outcomes
PRISMA: Preferred Reporting Items for Systematic Reviews and Meta-Analyses
SIV: seasonal influenza vaccine
Tdap: tetanus, diphtheria, pertussis
WHO: World Health Organization
