# Supplementary material for: Social Media as an Effective Provider of Quality-Assured and Accurate Information to Increase Vaccine Rates: Systematic Review
Source: J Med Internet Res. 2023 Dec 26;25:e50276. doi: 10.2196/50276 (PMC10777282; doi:10.2196/50276)
Supplement: Multimedia Appendix 2 [file jmir_v25i1e50276_app2.pdf]

Medline

Database:
Ovid MEDLINE(R) and In-Process, In-Data-Review & Other Non-Indexed Citations and Daily <1946 to May 29, 2023>

#	**Query**	**Results from 29 May2023**
1	exp Social media/ or (communication media* or discussion board* or social media* or online social network* or Facebook or Instagram or LinkedIn or Pinterest or Reddit or Renren or Sina Weibo or Snapchat or TikTok or Tumblr or Twitter or Vine or VK or VKontakte or WhatsApp or WeChat or YouTube).ti,ab,kw.	46,693
2	exp Vaccines/ or (vaccine acceptance or vaccine uptake or Vaccin* rate* or vaccination program* or vaccination coverage or vaccination campaign* or vaccination awareness or immuni?ation or immuni?ation coverage or immuni?ation program or health campaign* or health communication).ti,ab,kw.	358,383
3	exp Randomized Controlled Trial/ or randomized.ti,ab,kw.	930,029
4	1 and 2 and 3	98

exp Social media/ or (communication media* or discussion board* or social media* or online social network* or Facebook or Instagram or LinkedIn or Pinterest or Reddit or Renren or Sina Weibo or Snapchat or TikTok or Tumblr or Twitter or Vine or VK or VKontakte or WhatsApp or WeChat or YouTube).ti,ab,kw.

exp Vaccines/ or (vaccine acceptance or vaccine uptake or Vaccin* rate* or vaccination program* or vaccination coverage or vaccination campaign* or vaccination awareness or immuni?ation or immuni?ation coverage or immuni?ation program or health campaign* or health communication).ti,ab,kw.

exp Randomized Controlled Trial/ or randomized.ti,ab,kw.

1 and 2 and 3

https://ovidsp.ovid.com/ovidweb.cgi?T=JS&NEWS=N&PAGE=main&SHAREDSEARCHID=37idiAierdovl3SMLbzU2wXd7ciuNk6IxaNv4pNSG8IozQgAHXsw4fZB0AiUnS6UM

Embase

Database:
Embase Classic+Embase <1947 to 2023 May 29>

#	**Query**	**Results from 29 May 2023**

1	exp Social media/ or (communication media* or discussion board* or social media* or online social network* or Facebook or Instagram or LinkedIn or Pinterest or Reddit or Renren or Sina Weibo or Snapchat or TikTok or Tumblr or Twitter or Vine or VK or VKontakte or WhatsApp or WeChat or YouTube).ti,ab,kw.	69,172
2	exp Vaccines/ or (vaccine acceptance or vaccine uptake or Vaccin* rate* or vaccination program* or vaccination coverage or vaccination campaign* or vaccination awareness or immuni?ation or immuni?ation coverage or immuni?ation program or health campaign* or health communication).ti,ab,kw.	501,762
3	exp Randomized Controlled Trial/ or randomized.ti,ab,kw.	1,282,261
4	1 and 2 and 3	120

exp Social media/ or (communication media* or discussion board* or social media* or online social network* or Facebook or Instagram or LinkedIn or Pinterest or Reddit or Renren or Sina Weibo or Snapchat or TikTok or Tumblr or Twitter or Vine or VK or VKontakte or WhatsApp or WeChat or YouTube).ti,ab,kw.

exp Vaccines/ or (vaccine acceptance or vaccine uptake or Vaccin* rate* or vaccination program* or vaccination coverage or vaccination campaign* or vaccination awareness or immuni?ation or immuni?ation coverage or immuni?ation program or health campaign* or health communication).ti,ab,kw.

exp Randomized Controlled Trial/ or randomized.ti,ab,kw.

1 and 2 and 3

https://ovidsp.ovid.com/ovidweb.cgi?T=JS&NEWS=N&PAGE=main&SHAREDSEARCHID=2iycIYNaLBqiNm2d5k2D0sVKPaBfiv8TFWGa1geSWUY92gIiSDvl8o0goOzt3KLXo

PsycInfo

Database:
APA PsycInfo <1987 to May Week 22 2023>

#	Query	Results from 29 May 2023
1	exp Social media/ or (communication media* or discussion board* or social media* or online social network* or Facebook or Instagram or LinkedIn or Pinterest or Reddit or Renren or Sina Weibo or Snapchat or TikTok or Tumblr or Twitter or Vine or VK or VKontakte or WhatsApp or WeChat or YouTube).ti,ab.	34,345
2	exp Immunization/ or (vaccine acceptance or vaccine uptake or Vaccin* rate* or vaccination program* or vaccination coverage or vaccination campaign* or vaccination awareness or immuni?ation or immuni?ation coverage or immuni?ation program or health campaign* or health communication).ti,ab.	10,696
3	exp Randomized Controlled Trial/ or (randomized or RCT).ti,ab.	87,134

4	1 and 2 and 3	16

exp Social media/ or (communication media* or discussion board* or social media* or online social network* or Facebook or Instagram or LinkedIn or Pinterest or Reddit or Renren or Sina Weibo or Snapchat or TikTok or Tumblr or Twitter or Vine or VK or VKontakte or WhatsApp or WeChat or YouTube).ti,ab.

exp Immunization/ or (vaccine acceptance or vaccine uptake or Vaccin* rate* or vaccination program* or vaccination coverage or vaccination campaign* or vaccination awareness or immuni?ation or immuni?ation coverage or immuni?ation program or health campaign* or health communication).ti,ab.

exp Randomized Controlled Trial/ or (randomized or RCT).ti,ab.

1 and 2 and 3

https://ovidsp.ovid.com/ovidweb.cgi?T=JS&NEWS=N&PAGE=main&SHAREDSEARCHID=2iycIYNaLBqiNm2d5k2D0szLTZvKUltpcXlk481Gqz0ELG5A169KXzHDL64zgW0YI

Cinahl

S4	S1 AND S2 AND S3	Expanders - Apply equivalent subjects<br>Search modes - Boolean/Phrase	Interface - EBSCOhost Research Databases<br>Search Screen - Advanced Search<br>Database - CINAHL	30
S3	MH "Randomized Controlled Trials+" or TI(randomized) or AB(randomized)	Expanders - Apply equivalent subjects<br>Search modes - Boolean/Phrase	Interface - EBSCOhost Research Databases<br>Search Screen - Advanced Search<br>Database - CINAHL	306,661
S2	MH“Vaccines+” or TI(“vaccine acceptance” or “vaccine uptake” or “Vaccin* rate*” or “vaccination program*” or “vaccination coverage” or “vaccination campaign*” or “vaccination awareness” or immuni#ation or “immuni#ation coverage” or “immuni#ation program” or “health campaign*” or “health communication”) or AB(“vaccine acceptance” or “vaccine uptake” or “Vaccin* rate*” or “vaccination program*” or “vaccination coverage” or	Expanders - Apply equivalent subjects<br>Search modes - Boolean/Phrase	Interface - EBSCOhost Research Databases<br>Search Screen - Advanced Search<br>Database - CINAHL	69,751

	“vaccination campaign*” or “vaccination awareness” or immuni#ation or “immuni#ation coverage” or “immuni#ation program” or “health campaign*” or “health communication”)			
S1	MH“Social media” or TI(“communication media*” or “discussion board*” or “social media*” or “online social network*” or Facebook or Instagram or LinkedIn or Pinterest or Reddit or Renren or “Sina Weibo” or Snapchat or TikTok or Tumblr or Twitter or Vine or VK or VKontakte or WhatsApp or WeChat or YouTube) or AB(“communication media*” or “discussion board*” or “social media*” or “online social network*” or Facebook or Instagram or LinkedIn or Pinterest or Reddit or Renren or “Sina Weibo” or Snapchat or TikTok or Tumblr or Twitter or Vine or VK or VKontakte or WhatsApp or WeChat or YouTube)	Expanders - Apply equivalent subjects<br>Search modes - Boolean/Phrase	Interface - EBSCOhost Research Databases<br>Search Screen - Advanced Search<br>Database - CINAHL	34,960

Cochrane Central Register of Controlled Trials (CENTRAL)

Search Name:
Date Run: 04/03/2022 07:06:33
Comment:

ID Search Hits
#1 MeSH descriptor: [Social Media] explode all trees 238
#2 ("communication media*" or "discussion board*" or "social media*" or "online social network*" or Facebook or Instagram or LinkedIn or Pinterest or Reddit or Renren or "Sina Weibo" or Snapschat or TikTok or Tumblr or Twitter or Vine or VK or VKontakte or Whatsapp or Wechat or Youtube):ti,ab,kw 3289
#3 MeSH descriptor: [Vaccines] explode all trees 13796
#4 ("vaccine acceptance" or "vaccine uptake" or "vaccin* rate*" or "vaccination program*" or "vaccination coverage" or "vaccination campaign*" or "vaccination awareness" or immunization or "immunization coverage" or "immunization program"or "health campaign*" or "health communication"):ti,ab,kw 0
#5 MeSH descriptor: [Randomized Controlled Trial] explode all trees 119
#6 RCT:ti,ab,kw 32836

#7	#1 or #2	3289
#8	#3 or #4	1881664
#9	#7 or #8	1881664
#10	#7 and #8 and #9 in Cochrane Reviews	16
