# Supplementary material for: Social Media as an Effective Provider of Quality-Assured and Accurate Information to Increase Vaccine Rates: Systematic Review
Source: J Med Internet Res. 2023 Dec 26;25:e50276. doi: 10.2196/50276 (PMC10777282; doi:10.2196/50276)
Supplement: Multimedia Appendix 3 [file jmir_v25i1e50276_app3.docx]

| **#** | **Author** | **Year** | **Heading** | **Exclusion** |
| --- | --- | --- | --- | --- |
| 1. | Allington, D. et al. [24] | 2021 | Media usage predicts intention to be vaccinated against SARS-CoV-2 in the US and the UK | Not an RCT Wrong outcome |
| 2. | Arede, M. et al. [25] | 2019 | Combating vaccine hesitancy: teaching the next generation to navigate through the post truth era | Not an RCT Wrong outcome |
| 3. | Ateudjieu, J. et al. [26] | 2022 | Tracking Demographic Movements and Immunization Status to Improve Children's Access to Immunization: Field-Based Randomized Controlled Trial | Randomization of groups |
| 4. | Bethke, N et al. [27] | 2022 | A school-based educational on-site vaccination intervention for adolescents in an urban area in Germany: feasibility and psychometric properties of instruments in a pilot study | Cluster randomized No social media Wrong study design |
| 5. | Bonnevie, E. et al. [28] | 2020 | Using social media influencers to increase knowledge and positive attitudes toward the flu vaccine | Not an RCT |
| 6. | Bravo, C. et al. [29] | 2022 | Using social media listening and data mining to understand travellers’ perspectives on travel disease risks and vaccine-related attitudes and behaviours | Not an RCT Wrong outcome |
| 7. | Buller, D. B. et al. [30] | 2019 | Insights on HPV vaccination in the United States from mothers' comments on Facebook posts in a randomized trial | Attitudes to vaccines Wrong outcome |
| 8. | Buller, D. B. et al. [22] | 2021 | Human Papillomavirus Vaccination and Social Media: Results in a Trial With Mothers of Daughters Aged 14–17 | Lack of control group |
| 9. | Buller, D et al. [31] | 2022 | Promoting Social Distancing and COVID-19 Vaccine Intentions to Mothers: Randomized Comparison of Information Sources in Social Media Messages | Lacked a control group |
| 10. | Chen, S. et al. [32] | 2022 | Animated, video entertainment-education to improve vaccine confidence globally during the COVID-19 pandemic: an online randomized controlled experiment with 24,000 participants | Not Social Media Wrong outcome |
| 11. | Cutrona, S. L. et al. [33] | 2018 | Improving rates of outpatient influenza vaccination through EHR portal messages and interactive automated calls: a randomized controlled trial | Not Social Media Wrong outcome |
| 12. | Dempsey, A. et al. [34] | 2015 | Interventions to improve adolescent vaccination: what may work and what still needs to be tested | Not an RCT Wrong outcome |
| 13. | Dennis, A. S. et al. [35] | 2021 | Assessment of the Effectiveness of Identity-Based Public Health Announcements in Increasing the Likelihood of Complying With COVID-19 Guidelines: Randomized Controlled Cross-sectional Web-Based Study | Not about vaccines Wrong outcome |
| 14. | Ennab, F. et al. [36] | 2022 | Implications of social media misinformation on COVID-19 vaccine confidence among pregnant women in Africa | Not an RCT Wrong outcome |
| 15. | Fadda, M. et al. [37] | 2018 | Evaluation of a Mobile Phone–Based Intervention to Increase Parents’ Knowledge About the Measles-Mumps-Rubella Vaccination and Their Psychological Empowerment: Mixed-Method Approach | Not Social Media Wrong outcome |
| 16. | Featherstone, J. D. et al. [38] | 2020 | Feeling angry: the effects of vaccine misinformation and refutational messages on negative emotions and vaccination attitude | Not an RCT |
| 17. | Folkvord, F. et al. [39] | 2022 | Effect of Source Type and Protective Message on the Critical Evaluation of News Messages on Facebook: Randomized Controlled Trial in the Netherlands | Wrong outcome |
| 18. | Bian J. et al. [40] | 2019 | Social Media-Based Health Interventions: Where Are We Now? | Not an RCT Wrong outcome |
| 19. | Habib, G. L. et al. [41] | 2023 | The importance of cultural tailoring of communicators and media outlets in an influenza vaccination awareness campaign: a digital randomized trial | Cultural tailored messages |
| 20. | Jiang, Q. et al. [42] | 2022 | Social Media for Health Campaign and Solidarity Among Chinese Fandom Publics During the COVID-19 Pandemic | Not about vaccines |
| 21. | Kearney, M. et al. [43] | 2020 | Examining the #HPV vaccine on Instagram: An analysis of post context, imagery, and sentiment | Not an RCT |
| 22. | Kim, S. J. Et al. [44] | 2022 | Countering Antivax Misinformation via Social Media: Message-Testing Randomized Experiment for Human Papillomavirus Vaccination Uptake | Wrong outcome |
| 23. | Kim, S. C. et al. [45] | 2020 | An Eye Tracking Approach to Understanding Misinformation and Correction Strategies on Social Media: The Mediating Role of Attention and Credibility to Reduce HPV Vaccine Misperceptions | Not about vaccines |
| 24. | Kolff, C. A. et al. [46] | 2018 | The use of technology to promote vaccination: A social ecological model based framework | Not an RCT Wrong outcome |
| 25. | Kriss, J. L. et al. [47] | 2017 | Evaluation of two vaccine education interventions to improve pertussis vaccination among pregnant African American women: a randomized controlled trial | Not Social Media Wrong outcome |
| 26. | McRee, A. L. et al. [48] | 2018 | Effects of a pilot randomized controlled trial of a web-based HPV vaccination intervention for young gay and bisexual men: The outsmart HPV project | Not Social Media Wrong outcome |
| 27. | Myrick, J. G. et al. [49] | 2021 | A Mixed Methods Inquiry into the Role of Tom Hanks' COVID-19 Social Media Disclosure in Shaping Willingness to Engage in Prevention Behaviors | Not an RCT Wrong outcome |
| 28. | Parkkonen, J. et al. [50] | 2021 | What are the most effective office-based strategies to increase vaccine uptake among vaccine-hesitant parents? | Not an RCT Wrong outcome |
| 29. | Patel, A. et al. [51] | 2014 | Staying on track: A cluster randomized controlled trial of automated reminders aimed at increasing human papillomavirus vaccine completion | Randomization of centers |
| 30. | Petkovic, J. et al. [52] | 2021 | Behavioral interventions delivered through interactive social media for health behavior change, health outcomes, and health equity in the adult population | Not an RCT Wrong outcome |
| 31. | Robichaud, P et al. [21] | 2012 | Vaccine-critical videos on YouTube and their impact on medical students’ attitudes about seasonal influenza immunization: A pre and post study | Lack of control group |
| 32. | Salmon, D. A. et al. [53] | 2019 | MomsTalkShots: An individually tailored educational application for maternal and infant vaccines | Not Social Media Wrong outcome |
| 33. | Shoup, J. A. et al. [23] | 2014 | Development of an interactive social media tool for parents with concerns about vaccines | Not an RCT Wrong outcome |
| 34. | Si, M. et al. [54] | 2022 | Effect of an IMB Model-Based Education on the Acceptability of HPV Vaccination Among College Girls in Mainland China: A Cluster RCT | Not Social Media Wrong outcome |
| 35. | Stockwell, M. S. et al. [55] | 2013 | Utilizing health information technology to improve vaccine communication and coverage | Not an RCT Wrong outcome |
| 36. | Suzuki, Y. et al. [56] | 2021 | Effect of a Brief Web-Based Educational Intervention on Willingness to Consider Human Papillomavirus Vaccine for daughters and sons in Japan: A Randomized, Controlled Trial | Not Social Media Wrong outcome |
| 37. | Tjaden, J. et al. [57] | 2022 | Experimental evidence on improving COVID-19 vaccine outreach among migrant communities on social media | Wrong study design Lacked a control group |
| 38. | Wang, Q. et al. [58] | 2021 | The use of Web-based interactive technology to promote HPV vaccine uptake among young females: a randomized controlled trial | The intervention is a web-site |
| 39. | Welch, V. et al. [59] | 2018 | Interactive social media interventions for health behavior change, health outcomes, and health equity in the adult population | Not an RCT Wrong outcome |
| 40. | Wilson, K. et al. [60] | 2014 | Opportunities for utilizing new technologies to increase vaccine confidence | Not an RCT Wrong outcome |
| 41. | Witus, L. S. et al. [61] | 2022 | A randomized controlled trial of a video intervention shows evidence of increasing COVID-19 vaccination intention | No social media |
| 42. | Yan, C. et al. [62] | 2021 | Comparing Public Sentiment Toward COVID-19 Vaccines Across Canadian Cities: Analysis of Comments on Reddit | Not an RCT Wrong outcome |
